# The use of videoconferencing with patients with psychosis: a review of the literature

**DOI:** 10.1186/1744-859X-10-14

**Published:** 2011-04-18

**Authors:** Ian R Sharp, Kenneth A Kobak, Douglas A Osman

**Affiliations:** 1 MedAvante Research Institute, Hamilton, NJ, USA; 2 Center for Psychological Consultation, Madison, Wisconsin, USA

## Abstract

Videoconferencing has become an increasingly viable tool in psychiatry, with a growing body of literature on its use with a range of patient populations. A number of factors make it particularly well suited for patients with psychosis. For example, patients living in remote or underserved areas can be seen by a specialist without need for travel. However, the hallmark symptoms of psychotic disorders might lead one to question the feasibility of videoconferencing with these patients. For example, does videoconferencing exacerbate delusions, such as paranoia or delusions of reference? Are acutely psychotic patients willing to be interviewed remotely by videoconferencing? To address these and other issues, we conducted an extensive review of Medline, PsychINFO, and the Telemedicine Information Exchange databases for literature on videoconferencing and psychosis. Findings generally indicated that assessment and treatment via videoconferencing is equivalent to in person and is tolerated and well accepted. There is little evidence that patients with psychosis have difficulty with videoconferencing or experience any exacerbation of symptoms; in fact, there is some evidence to suggest that the distance afforded can be a positive factor. The results of two large clinical trials support the reliability and effectiveness of centralized remote assessment of patients with schizophrenia.

## Introduction

Technological advances in recent years have made remote psychiatric assessment and treatment significantly more feasible. In particular, the increased availability and affordability of high-speed connections have made the use of videoconferencing (VC) a viable tool for interacting with patients remotely. There is a growing body of literature on telemedicine and the subfield of telepsychiatry. The initial thrust to develop these fields was prompted by attempts to meet demands for mental health services with underserved and difficult-to-serve populations (for example, rural areas, prisons). For instance, extensive telepsychiatry networks in rural Australia and Canada were created to improve access to mental health services. More recently, other VC applications such as the training of mental health professionals and centralized ratings in clinical trials have grown out of this rapidly expanding field. As telepsychiatry evolves, a broader range of patient populations can be served through this medium.

Several factors make the assessment and treatment of psychosis particularly well suited for VC. For one, as psychotic patients are often hospitalized, VC allows patients to be connected with specialists without need for travel. Assessment and treatment using VC is also a potential solution for patients with psychosis living in remote or underserved areas where there is a shortage of specialists. As a tool in clinical research, VC makes it possible to use centralized remote expert raters who are able to remain blind to study design and conditions, therefore decreasing rater bias and improving inter-rater reliability and interview quality [1].

The hallmark symptoms of psychotic disorders might lead one to question the feasibility of using VC with this patient population. For example, are acutely psychotic patients generally willing to be interviewed remotely by videoconference? Does videoconferencing exacerbate delusions, such as delusions of reference? Are scores on symptom severity rating scales and diagnoses obtained remotely by videoconference equivalent to ratings and diagnosis performed face to face, given the complex nature of the disorder and the importance of non-verbal signs, such as negative symptoms? Is treatment conducted remotely by videoconference as effective as treatment conducted in person? Are evaluations conducted over VC sensitive enough to distinguish active drug from placebo in clinical trials?

In the present work we attempted to provide answers to these questions by conducting a thorough review of the literature. For the purposes of this review, videoconferencing refers to an interactive video connection between two sites. This primarily includes two-way videoconferencing using monitors or computers connected over telephone lines (for example, integrated services digital network (ISDN)), public internet connections, or private networks, but may also include the use of closed-circuit televisions, especially in older studies, for example, Dongier *et al*. [2]. An important variable in evaluating VC studies is bandwidth. In videoconferencing, bandwidth refers to the speed of transmission of data between two points, typically expressed in kilobits per second (kbps). The studies reviewed had a range of bandwidths from narrow (for example, 33 kbps) to broad (for example, 384 kbps). As a rule of thumb, the higher the bandwidth, the better the quality of audio and video. The current VC industry standard bandwidth is 384 kbps. A second important variable in understanding the quality of VC is frame rate. Frame rate refers to the number of frames presented on a monitor, typically expressed in frames per second (fps). The higher the frame rate the better motion is presented in video. A speed of 30 fps provides a continuous picture similar to television quality and generally requires 384 kbps transmission [3]. As found in other reviews [4], this variable was frequently not reported.

## Methods

We reviewed the Medline, PsychINFO, and the Telemedicine Information Exchange databases for literature on videoconferencing and psychosis. We used the following key words: telemedicine, telepsychiatry, televideo, videoconferencing, video conferencing, video and schizophren*, schizoaffective, psychotic, and psychosis. No date restrictions were used. Articles relevant to the use of videoconferencing with persons with psychosis were included in this review. We also reviewed reference sections for additional relevant articles. The literature search was completed in September 2010.

We present our findings in the following categories: clinical interventions (7 articles); assessment (12 articles); satisfaction and acceptance (12 articles); and clinical trials (2 articles). The small number of articles precluded quantitative analysis, but careful review allowed for qualitative assessment, which is the approach of the present manuscript. Please see Additional file 1 for a brief description of each of the references included in the review.

## Results

### Clinical interventions

The majority of articles written about the clinical utility of VC with psychotic patients have been retrospective reports of programs that provided services to remote areas. Dwyer [5] described a series of programs and general clinical uses of a closed circuit interactive television (IATV) system set up, a precursor to VC, between Massachusetts General Hospital and a medical station in Boston. Approximately 5% of all those seen on IATV had severe psychiatric disorders. The author admitted that he 'approached the use of television to interview psychiatric patients with considerable negative prejudice, believing that the degree of personal contact with the patient would be limited and that many of the skills that are useful in a psychiatric interview would be diminished or lost. I was delightfully surprised to discover that this was not true'. The author reported that approximately 30 psychiatrists and an equal number of psychiatric residents and medical students used the television system, and all responded positively to their experiences. The author suggested that, for some patients, communication with a psychiatrist by means of IATV was 'easier' than contact in the same room. It was suggested that this is especially true of patients with schizophrenia. The author also reported that a number of patients with delusions were interviewed and none incorporated the television into his or her distorted thinking.

Graham [6] discussed a program designed exclusively for chronically mentally ill individuals. The project was called APPAL-LINK, the Southwestern Virginia Telepsychiatry Project, and provided services by connecting hospital psychiatrists to patients at two rural community mental health centers. The author reported that 39 patients with a wide variety of diagnoses were followed through the initial 6 months of operation. The majority of these patients had a major psychotic illness such as schizophrenia, bipolar disorder, or schizoaffective disorders. The author reported that the availability of telepsychiatry consultation for crisis intervention led to a decrease in hospitalizations and no significant adverse effects were reported. It was also noted that patients and psychiatrists adjusted well to the VC interaction and that the program provided evidence that VC is 'a safe, effective, and useful method for the outpatient treatment of chronically mentally ill patients'.

In a report of a larger program involving the use of telemedicine, Zaylor [7] reviewed the history of VC at the University of Kansas Medical Center. At the time the article was written, Zaylor reported that the Telepsychiatry Service of the Department of Psychiatry and Behavioral Sciences was providing services to 18 locations throughout the state. One of the programs described was a group composed of six patients with either schizoaffective disorder or schizophrenia, which met monthly over VC for nearly 3 years. Anecdotally, Zaylor reported that many of the patients' conditions improved and stabilized over time. Other programs reviewed in the article included the use of VC to provide psychiatric services to inmates in a rural county jail clinic and to residents in a rural group home for the chronically mentally ill. Zaylor stated that patients in each program accepted the technology readily and quality of care was not diminished.

In another study, Zaylor [8] completed a retrospective review of patient records comparing clinical outcomes of patients seen by IATV and those seen in person. The IATV condition consisted of PC-based VC equipment with a bandwidth speed of 128 kbps. A global assessment of functioning (GAF) score was generated for each patient in both groups at the initial visit and at subsequent visits, including at 6 months. A total of 49 patients diagnosed with either major depression or schizoaffective disorder were included. No significant difference was found in the percentage change in GAF scores between the two groups, suggesting that clinical outcomes were not negatively impacted by the use of IATV. The authors noted that patients in the IATV group had a better attendance rate and follow-up visits took less than half the time compared with in-person visits. This was viewed as an indication that IATV was an acceptable and efficient method of providing psychiatric services.

Doze and colleagues [9] reported preliminary results of a 9-month pilot project in Alberta, Canada, which used VC to connect a psychiatric hospital to mental health clinics in five rural hospitals. Patients were most commonly referred for assistance with a diagnosed disorder or to establish a diagnosis, but were also referred for behavior management, medication consultation, patient education, follow-up after discharge, and preadmission screening. A total of 109 telepsychiatry consultations were completed with 90 patients, 8 of whom were diagnosed with schizophrenia. Like many of the studies in this review, the authors focused on the usage of telepsychiatry including cost analysis and opinions about its use rather than measured clinical outcomes. However, the authors noted positive anecdotal results, including indications that the telepsychiatry project led to the avoidance of hospitalization for some patients as well as reduced stigma for patients who visited an acute care facility rather than a mental health clinic.

D'Souza [10] documented a telemedicine service in rural Australia developed to treat acute psychiatric inpatients in their local hospitals in order to reduce the need for these patients to be transferred to a psychiatric facility farther away. In all, 28 patients were included in the report; 31% were diagnosed with schizoaffective disorder, 11% were diagnosed with schizophreniform psychosis, and 4% were diagnosed with delusional disorder. The Brief Psychiatric Rating Scale 24 (BPRS-24) [11] was administered by both a rater familiar with the patient and a naïve rater at intake and 4 weeks after discharge. Results indicated a significant improvement in the mean total BPRS-24 scores from intake to follow-up for both raters and inter-rater reliability for the BPRS-24 was good. The authors conclude that these findings support the use of VC in the evaluation of clinical outcomes in treatment.

Kennedy and Yellowlees [12] examined clinical outcomes in the use of VC with 124 patients entering mental health treatment in rural Queensland, Australia. All patients were offered the option of being treated by a psychiatrist using a VC system at 128 kbps and 32 patients (3 of whom were diagnosed with psychotic disorders) chose the VC option. All patients were assessed when entering treatment and then 12 months later. The authors reported significant improvement from pre-assessment to post-assessment as measured by the Health of the Nation Outcome Scale (HoNOS), a clinical outcome scale [13] and the Mental Health Inventory (MHI), a self-report scale of outcome or progress over time [14], but no significant differences were found between the VC and in-person conditions. The authors concluded that there was no degradation in quality of outcome with the use of VC.

Published reports on clinical interventions delivered using VC have shown that patient care via VC is generally equivalent to in person. Further, the advantages of VC have been outlined and include less need for patients and professionals to travel, reduction in hospitalizations, and improvement in reaching patients in rural and challenging settings. There is virtually no evidence that VC has a negative impact on rapport, especially in more recent reports where technology is less likely to be a barrier. Additionally, there is evidence that some patients with psychosis prefer receiving clinical services via VC to in person. Children especially tend to be more forthcoming with telepsychiatry [15]. Most of the clinical intervention reports reviewed were qualitative accounts of clinical work being performed with patients with psychosis via VC. While these papers provide strong evidence of the feasibility of VC with patients with psychosis, additional empirical research (for example, treatment outcome studies) is needed.

### Assessment

Published reports of assessment of psychosis using VC primarily fell into two broad categories: uncontrolled case reports of clinical evaluations, and reports of systematic evaluations of objective instruments of schizophrenia. We also include a published report evaluating rater training with a psychosis scale using live interviews conducted via VC.

Hyler *et al*. [16] conducted a meta-analysis of studies comparing psychiatric assessment via VC to in person. Although not specific to psychosis, they concluded that objective assessments delivered via VC were equivalent to in person in both accuracy and satisfaction.

One of the earliest studies related to VC and assessment involved using closed circuit television (CCTV), a precursor of modern day VC, to conduct psychiatric evaluations. Dongier and colleagues [2] compared psychiatric interviews conducted using CCTV to a control group in which interviews were conducted in person. The study included inpatients and outpatients from a range of diagnostic categories including schizophrenic psychoses (27%), schizophreniform psychoses (6%), and paranoid states (2%). The authors concluded that 'even schizophrenics with ideas of reference including T.V. (example: being talked about on public programs) accepted the CCTV interaction very well and no exacerbation of their delusions was observed'.

In a later description of psychiatric evaluations using VC, Yellowlees [17] presented two case reports in which urgent psychiatric assessments for two psychotic patients were conducted using VC. Without the use of VC, the patients would have had to travel to a psychiatric hospital 800 km away. The author noted that one of the patients with delusional symptoms reported ideas of reference from the television prior to the interview, but accepted the interview and interaction with the assessor as real.

Ball and colleagues [18] presented data from a more controlled study of the use of VC for assessment of psychiatric patients. The authors administered the Folstein Mini-Mental State Examination (MMSE) [19] to 11 patients from an acute psychiatric ward (6 patients were diagnosed with schizophrenia). Each patient was interviewed both in person and over VC. In person assessments were compared to a computer-based low-cost videoconferencing (LCVC) system. The scores between modalities were highly correlated leading the authors to conclude that the MMSE may be reliably performed with patients using LCVC. However, the authors noted that one patient did not complete the second assessment because he developed a delusional belief that the testing was part of a police plot to incriminate him. This appeared unassociated with the LCVC as he had completed that portion (that is, VC) and refused the in person interview.

Several studies have reported on the use of VC using the BPRS [20]. Salzman *et al*. [21] reported the use of VC in administering this instrument to evaluate severely ill inpatients. After establishing inter-rater reliability on the BPRS (0.93) by using in person interviews with patients in the hospital, six psychotic patients were rated using videoconferencing. Patients were simultaneously rated by a psychiatrist via videoconferencing and a psychiatrist who was on site. The reported inter-rater reliability was 0.92. The authors noted that the only frequent rating disagreement was on a self-neglect item and they concluded that some patients' self-neglect was difficult to observe via VC. However, a limitation of this conclusion is that the authors did not report data on the quality or speed of the VC equipment and connection. The patients reportedly enjoyed using VC. The authors concluded that these results add to previous research suggesting that VC is useful in the evaluation of psychotic patients.

Baigent and colleagues [22] also used the BPRS when comparing VC using ISDN connections at 128 kbps to in person interviews. In addition to the BPRS, the authors used a semi-structured clinical interview to generate *Diagnostic and Statistical Manual of Mental Disorders*, 4th edition (DSM-IV) diagnoses. The 2 psychiatrists conducted the assessments with 63 subjects (51% of whom had a diagnosis of schizophrenia). Interviews were conducted in one of three conditions: the interviewer and observer in the same room as the patient, the interviewer connected to the patient via VC and the observer in the same room as the patient, or both the interviewer and the observer connected to the patient via VC. Inter-rater reliability for BPRS total score in the three conditions was 0.54, 0.51, and 0.80, respectively. The authors reported that reliability of diagnoses was equivalent in the three conditions (0.85, 0.69, 0.70, respectively) and concluded that 'much of the 'psychiatry' is not lost in 'telepsychiatry'.'

Zarate and colleagues [23] also assessed the reliability of the BPRS in addition to the Scales for the Assessment of Positive/Negative Symptoms (SAPS/SANS) [24] in a sample of 45 patients with a DSM-IV diagnosis of schizophrenia. Assessments were conducted either in person or via VC (at either 128 kbps or 384 kbps). Assessments in the in person condition were conducted with two raters in the same room as the patient with one conducting the interview and the other rating the patient's responses. In the VC condition, one rater conducted the interview remotely and the other rater scored the patient's responses while sitting in the same room as the patient. Results indicated good overall inter-rater reliability on total BPRS scores with both 384 kbps (intraclass correlation coefficient (ICC) = 0.90) and 128 kbps (ICC = 0.84) connections. Excellent reliabilities were also found on the positive symptoms scale (SAPS ICC = 0.97 for both low and high bandwidths). Higher reliabilities were found with the 384 kbps connection (0.85) vs. the 128 kbps connection (0.67) on the SANS. Given that several specific negative symptoms of schizophrenia rely heavily on non-verbal cues, it is understandable that the higher bandwidth would improve agreement on these symptoms. Both raters and patients had high rates of acceptance of the VC condition with patients in the high bandwidth group being more likely to prefer it to live interviews than those in the low bandwidth group.

In another study examining reliability at different connection speeds, Matsuura and colleagues [25] reported the reliability of the BPRS administered in person or via one of two resolutions of videophone (128 kbps and 384 kbps). In all, 17 subjects were included (9 healthy nursing students and 8 outpatients, 2 of whom had a diagnosis of schizophrenia). The study had three conditions: an in person condition where two raters were in the same room as the patient, a low-resolution VC interview condition where a rater was linked to the patient with a TV phone at 128 kbps and an observer was in the same room as the patient, and a similar condition with a high-resolution TV phone at 384 kbps. Interclass correlation coefficients were very high for all three conditions (0.965, 0.987, 0.996, respectively) and did not differ significantly by condition. Additionally, 80% of the outpatients stated they preferred the VC interview.

Chae and colleagues [26] used a similar methodology to Matsuura and colleagues in a pilot study to evaluate a VC system connected over an ordinary telephone network at 33 kbps. A total of 30 patients with schizophrenia were administered the BPRS (15 using the VC system and 15 in person). Agreement on total BPRS score for the telemedicine group was significantly higher than that of the in person group. However, reliability on the anxiety subscale was very low for the telemedicine group. The authors suggested that the limited image processing capability of the system used may have made it difficult to conduct a detailed analysis of these specific symptoms. Overall, the authors concluded that the low-bandwidth VC system appeared to be as reliable as higher-bandwidth ISDN systems used in previous studies.

Yoshino and colleagues [27] assessed the reliability of the BPRS in 42 patients diagnosed with chronic schizophrenia. Patients were interviewed using videoconferencing with either narrow bandwidth (128 kbps) or broadband (2 Mbps) and compared to an in person interview using test-retest method with no longer than 4 days between the independent interviews. The authors found no significant difference in intraclass correlation coefficients for BPRS total score between the broadband condition (0.88) and the in-person condition (0.87). The ICC was significantly lower in the low bandwidth condition (0.44). It should be noted that the authors reported numerous problems in the narrow bandwidth condition including pauses in audio, problems with patients' speech clarity, highly distorted video images, poor rapport due to lack of eye contact, and almost total inability to observe facial expressions.

Lexcen *et al*. [28] conducted a study with 72 inpatients from the maximum security forensic unit of Central State Hospital in Petersburg, Virginia. All participants had DSM-IV Axis I diagnoses of severe mental illness; many were diagnosed with schizophrenia or psychotic disorder not otherwise specified (F J Lexcen, personal communication, 5 March 2007, Child Study and Treatment Center, Lakewood, WA). Participants were observed in one of three conditions. The first condition entailed in person administration of the BPRS with observation via video conferencing. The second condition involved administration by VC and observation by an in person rater. In the third condition, both administration and observation occurred in person. Correlations for total scale scores for the BPRS were in the good to excellent range (0.69 to 0.82). The results for the items of the BPRS were consistent with previous studies that found good to excellent reproducibility in experimental conditions using VC. The authors summarized that their results confirmed previous findings of the use of the BPRS for evaluations conducted via VC.

Kobak *et al*. reported on a National Institute of Mental Health (NIMH)-funded pilot study conducted to evaluate the effectiveness of training raters remotely by VC to administer the Positive and Negative Syndrome Scale (PANSS) [29]. The training involved two components: didactic training delivered via CD-ROM, and applied training delivered through live remote observation of trainees conducting the PANSS via VC. An expert trainer observed the interview and provided individual feedback immediately after the session via VC on the trainees' scoring accuracy and clinical interview skills using the Rater Applied Performance Scale (RAPS) [30]. Pre-training and post-training interviews were videotaped and evaluated by a panel of blinded experts to evaluate whether the training resulted in improvement in the trainees' clinical skills and scoring accuracy. In all, 12 trainees with no prior PANSS experience participated in the study. Results found a significant improvement in trainees' conceptual knowledge and an improvement in trainees' clinical skills (as determined by the RAPS scale). Interestingly, the didactic training (that is, CD-ROM) alone did not improve the trainees' clinical skills; these only improved following the remote video sessions. The agreement in scoring between the trainee and blinded expert (ICC) improved from r = 0.19 prior to training (*P *= 0.248) to r = 0.52 after training (*P *= 0.034). The results of this study are promising for the use of VC in the remote training of raters in schizophrenia.

Based on the studies reviewed, patients with psychosis can be reliably interviewed and evaluated via VC, including using symptom severity scales (for example, BPRS) and diagnostic, clinical, and psychiatric interviews. The reviewed findings suggest that higher bandwidth connections improve reliability and the ability to evaluate non-verbal and negative symptoms. At higher bandwidths, inter-rater reliability with VC is generally equivalent to in person. Additionally, VC can be used effectively to train raters in the administration of psychosis scales.

### Safety issues

The issue of patient safety has been raised when using VC for remote assessment and intervention with psychotic patients. The American Telemedicine Association has issued a set of practice guidelines for the emergency management of patients when using VC in telepsychiatry [31,32]. These guidelines require that a protocol be established for dealing with psychiatric emergencies when conducting any telepsychiatry procedure. Recommendations are provided in three main areas: (a) administrative issues, including requiring clinicians to conduct a site assessment to obtain information on local regulations and emergency resources, and having an emergency protocol in place that clearly specifies the procedures, roles, and responsibilities in cases of psychiatric emergencies; (b) legal issues, requiring clinicians to be familiar with local civil commitment regulations and have arrangements in place with local staff to initiate and assist in this regard; and (c) general clinical issues, including being aware of how clinicians' perception of diminished control in the clinical encounter compared to in person interaction might impact their interactions with the patient, and the need to be aware of the impact the telepsychiatry interaction might have on local site staff. With these safeguards in place, patient safety has not been reported as an issue when using VC with psychotic patients. In fact, it has been reported that the physical distance afforded by telepsychiatry has allowed patients to express strong affects that may have led to premature termination of in person sessions [32]. Nonetheless, these guidelines are relatively new and still evolving, and require ongoing examination and refinement.

### Satisfaction and acceptance

Many of the studies mentioned previously looking at the use of telepsychiatry in assessment and clinical outcomes also included measures of patient satisfaction. The overall results have been largely positive. Zarate and colleagues [23] asked patients and raters to complete post-interview evaluation and satisfaction questionnaires comparing their VC interview to in person interviews they have had in the past (from 'much below average' to 'much better than average'). A majority of patients rated the VC experience as 'above average', with patients in the higher bandwidth condition being more likely to prefer them to in person interviews. Raters endorsed comfort, ease of expressing one's self, and usefulness of VC as either 'average' or 'above average' as compared to a typical in person interview. Graham [6] indicated that patient acceptance of VC for healthcare delivery was almost universally positive with more than 90% of patients giving positive ratings on the satisfaction survey as it related to the VC process and treatment received. Similarly, in the Baigent *et al*. [22] study mentioned earlier, more subjects reportedly found interviews via VC moderately enjoyable to very enjoyable compared to the in person interviews. A majority of participants reported that they would be happy to have VC interviews or would even prefer them to seeing a psychiatrist in their hospital rooms.

Doze *et al*. [9] included data related to patient satisfaction in their telepsychiatry pilot project. The authors noted that patients were satisfied with and accepted the overall experience of using VC for psychiatric services. Perceived benefits noted by patients included reduced travel time; decreased stress from traveling to appointments; decreased absence from work for both patient and family; more immediate access to a psychiatrist; feelings of confidentiality and privacy; more patient choice and control; improvement in quality of life; and potential for clinical improvement without hospitalization. Perceived disadvantages noted by patients included feeling that their interaction with the psychiatrist was impersonal and the potential for less sensitivity in interviews. The authors noted that there was a strong preference for the use of VC rather than waiting for a consultation or traveling to see a psychiatrist, but patients were split as to whether they would rather use telepsychiatry than see a psychiatrist in person. Perceived benefits of VC noted by participating psychiatrists included the ability to see patients before their symptoms became more severe, to educate local providers, and to reduce amount of unproductive time that could now be used in psychiatric consultation.

In the study examining reliability at different connection speeds mentioned previously, Matsuura and colleagues [25] found that 80% of outpatients preferred telepsychiatry to in person interaction. The authors stated that many of the subjects reported that they could easily relate to the consultants and address problems without difficulty. One patient reported that the sound/picture delay was disturbing but no one reported dissatisfaction with the interview. Many patients reported that they would be happier having VC sessions at home to save time and effort.

Using a similar design, Chae and colleagues [26] asked patients to rate comfort level during the interview, ability to express themselves, quality of the interpersonal relationship, and usefulness of the interview. Total acceptance scores were higher in the VC condition than in the in person condition, although this difference was not statistically significant. Patients' acceptance of the VC interview, in terms of comfort, ease of self-expression, quality of interpersonal relationship and usefulness, was good in most cases. The average acceptance score was nearly twice as high in the telemedicine group as in the in person group. Patients tended to feel more comfortable in the in person condition, but more at ease with expressing themselves in the VC condition. The authors concluded that in many cases the VC condition was better accepted by patients and suggested that it might be viewed as less threatening than being in the same room in close proximity.

As part of his clinical outcome study, D'Souza [10] asked patients to rate their satisfaction with the service and the use of VC. The patients reportedly expressed high rates of satisfaction with both. Over 81% of patients said that they would use the service again; 88.8% reported high satisfaction with the VC practitioner; 70% were satisfied with receiving a prescription via VC; and 67% were satisfied with confidentiality. However, it should be noted that 26% of patients expressed some dissatisfaction, but the sources of the dissatisfaction were not specifically elaborated on in the report.

Ball and colleagues [33] compared the process and outcome of clinical tasks in an acute psychiatric unit using four different communication modes: in person, telephone, hands-free telephone, and a low-cost videoconferencing system (LCVC). Six doctors and six patients (three with schizophrenia and one with paranoid disorder) were included in the study. The authors report that the VC condition was positively received by both patients and doctors. However, some problems were observed. For instance, some patients found it irritating when the doctor leaned forward and only the top of his head was visible. One patient reportedly felt unable to talk about sexual delusions over the VC, although she felt comfortable discussing it in the other conditions.

Mannion and colleagues [34] presented results from a pilot project in which they used a PC-based VC system (384 kbps) to facilitate emergency consultations between patients on an Irish island and a psychiatrist on the mainland. Over an 8-month period, two patients diagnosed with schizophrenia were evaluated. The authors report that the patients were comfortable with the technology and stated that the system was not a barrier to the establishment of rapport. Additionally, all health professionals who used the link reportedly found it satisfactory. The authors concluded that the VC was acceptable and satisfactory for both patients and staff.

Stevens *et al*. [35] also conducted a pilot study of patient and clinician satisfaction with VC that included 19 patients with psychosis and 21 non-psychotic patients. Subjects were randomly assigned to either a VC or in person condition where they were assessed by psychiatrists during 90-minute unstructured interviews that were intended to generate *Diagnostic and Statistical Manual of Mental Disorders*, 3rd edition - revision (DSM-III-R) diagnoses and treatment recommendations. Following each interview, the participant and psychiatrist both completed the California Psychotherapy Alliance Scale [36], a self-report scale to assess ability to work together and develop rapport and the Interview Satisfaction Scale, a scale created for the study designed to assess acceptability of the interview modality. There were no differences on the patient-rated and clinician-rated alliance scale or the patient-rated satisfaction scale between modalities. There was a significant difference on the therapist version of the satisfaction scale with the psychiatrists tending to rate the VC interviews less favorably than the in person interviews; however, overall satisfaction with VC was still positive.

Magaletta *et al*. [37] examined prison inmates' satisfaction with VC consultations. A total of 75 patients, 17 with diagnoses of 'Schizophrenia and Other Psychotic Disorders', completed at least 1 questionnaire assessing their satisfaction with receiving psychiatric consultation via VC. Patients reported satisfaction with the consultation process, more comfort with the process over time, and a willingness to return for follow-up. A majority of the participants (81%) rated treatment positively, reported that they would come back to be seen by a doctor using VC (83%) and would recommend VC consultations to other inmates (71%). When looking at satisfaction ratings by time point, the results indicated that the participants' perceptions of the VC consultations became more positive over time. Participants with thought disorders had positive perceptions of the VC consultations and reported a higher level of satisfaction compared to in person treatment than did a group of inmates with affective disorders. The authors provided two examples of patients with thought disorders. One patient had consistently expressed delusions of reference from the TV in his housing unit. Despite hesitation on the part of the authors to include this patient in a VC consultation, they proceeded and found the only comment he made was 'See, I told you the television talks to me!'. They concluded that the patient's delusional system was not altered as a result of treatment using VC and that although the use of VC did not exacerbate his delusion, it may have reinforced it. The second example involved a patient with schizophrenia who felt that seeing his picture on the screen (because of a picture-in-picture option where the patient sees a small image of himself in addition to the remote image) confirmed his preexisting delusion that he had an impostor, leading the authors to discontinue the use of picture-in-picture. Despite these interactions between the technology and the delusional systems of several patients, the authors expressed that the patients were still able to receive sound treatment. The article offered possible explanations for the positive perceptions presented by thought-disordered patients. One explanation is that thought-disordered individuals are overstimulated in social and interpersonal relationships and the 'distance' accorded by VC serves to reduce their anxiety and help them feel more comfortable. Further, the structured and constrained nature of the VC environment also serves to lessen anxiety.

Mielonen *et al*. [38] conducted a study of inpatient care-planning consultations using VC with 14 patients with psychosis and their family members. Healthcare providers and patients and their relatives completed questionnaires of satisfaction and acceptance after each session. In all, 47% of the healthcare providers rated videoconferencing to be 'as good a form of consultation as a conventional meeting', 48% considered it to be 'almost as good', and only one person (4%) felt that it was notably inferior. The preference for VC was strong with most respondents preferring to have the next session conducted in the modality: 86% of the healthcare personnel, 84% of the patients and 92% of the relatives. The reduced need for traveling by the participants and the ease and speed of the consultations were cited as the most important reasons for preferring VC. Most of the respondents rated the content of the consultation and the interaction in the videoconference as excellent or good and the technical quality of the VC consultations as good or moderate.

In summary, most published reports show clearly that both patients and clinicians have high levels of acceptance and satisfaction with VC, often rating it similarly to in person, and in a number of cases rating it more favorably. There is some evidence that patient ratings of satisfaction with VC increase over time. Additionally, similar to findings with clinical interventions and assessment, higher bandwidth is associated with better outcome with satisfaction and acceptance.

### Clinical trials

While VC has been used widely with patients with psychosis in clinical settings, its use in clinical research with this population has not been extensively explored, but appears to be gaining acceptance. Clinical trials evaluating new medications for schizophrenia and other psychiatric disorders have been faced with an increasing rate of failed trials [39]. Factors associated with clinician assessment, such as expectancy bias, enrollment pressure bias, poor inter-rater reliability, and poor interview quality, have been hypothesized to play a role in this increasing rate [40]. The use of VC enables a potential solution to these problems, by facilitating the use of off-site expert centralized raters. These raters are linked to the various study sites through videoconferencing or teleconferencing, and remotely administer the primary outcome measure to study patients during their regularly scheduled study visit. The use of centralized raters in clinical trials addresses several potential weaknesses associated with clinician ratings described above. Inter-rater reliability is improved by simply reducing the sheer number of raters involved (for example, a 30-site multicenter trial that employed 60 to 75 raters (that is, 2 or 3 raters per site) could be conducted with 8-10 centralized raters). Rigorous training and calibration procedures can be employed that are not logistically feasible with a larger group of raters at diffuse study sites. Enrollment pressure and bias are minimized, since centralized raters are divorced from the study site and blinded to the study visit number, study protocol, and entrance criteria. Blinding the rater to these factors also minimizes expectancy or other biases at later visits. Using a different rater each week minimizes the potentially confounding therapeutic impact of repeated assessment by the same clinician, as well as minimizing expectancy bias.

Two published clinical trials using centralized raters via videoconferencing were identified. Centralized raters were recently used in a large, phase II, multicenter trial evaluating a new antipsychotic medication for schizophrenia [41]. A total of 289 subjects from 35 sites were randomly assigned to 6 weeks of treatment with 1 of 2 doses of an experimental compound, active comparator (olanzapine), or placebo. Subjects were evaluated weekly using the PANSS by 1 of 18 centralized raters who were connected to the study site by high speed VC at 384 kbps. Different raters typically saw the patient at each visit. Raters were blinded to study visit and study protocol and were provided informant data. Data from the olanzapine and placebo arms were provided by the sponsor to examine the issue of the centralized raters' ability to detect a drug effect.

Centralized raters found a significant difference between olanzapine and placebo starting at week 1, and this difference continued to be significant throughout the study. At endpoint, the mean change for olanzapine-treated participants (14.4 points, SE = 2.43) was significantly greater than the mean change on placebo (2.95 points, SE = 2.43), *P *< 0.001. The mean effect size found at endpoint was 0.52. Internal consistency reliability was high, and remained high throughout the study. Scores at screening were normally distributed, and were not skewed towards the cutoff score, suggesting that little score inflation occurred. Overall, 1,993 remote PANSS assessments were completed by the 18 raters over the 13-month course of the study. No patient refused to be interviewed by VC, although some patients refused to participate in all of the study assessments. Of the 1,993 assessments, 2.2% experienced temporary interruption or an audio/visual quality issue. The issues were resolved and the interviews were completed. In 10 cases (0.3%) the interview could not be completed due to a technical issue and had to be rescheduled.

Centralized raters were also used for efficacy ratings in a randomized, double-blind, placebo-controlled, multicenter phase III trial of the safety and efficacy of three doses of paliperidone palmitate in adults with an acute exacerbation of schizophrenia [42]. All subjects at US-based sites were evaluated by centralized raters using the PANSS, Personal and Social Performance Scale (PSP), and the Clinical Global Impression - Severity scale (CGI-S) and were connected to the study site by high speed VC at 384 kbps. The overall study had positive findings with each of the three doses of the drug demonstrating statistically significant improvement on the primary efficacy measure (PANSS total scores), and the two higher doses showing significant improvement with PSP and CGI-S scores. This study provides further evidence of the effectiveness of using VC as a tool for assessing participants in clinical trials. There has been rapid growth of adoption of centralized raters in clinical trials and there are currently several additional trials underway.

## Conclusions

Although there is still a paucity of controlled outcome research comparing VC to standard in person care, reports of assessment and treatment via VC have been overwhelmingly positive. Findings generally indicate that patient care via VC is equivalent to in person, but also offers numerous advantages. For example, reports indicate that the use of VC has led to a reduction in the need for patients and professionals to travel, a reduction in hospitalizations, and improvement in reaching patients in rural and difficult settings (for example, prisons), all leading to improved, more efficient care. There is little evidence that VC has a negative impact on rapport, although in some older studies comparing VC to in person, patients and clinicians preferred in person. This finding was generally attributed to poor video quality found with older technology. This preference is not evident in more recent research. In more recent studies [25,26], patients overwhelmingly preferred VC to in person.

Research and clinical work to date indicate that clinical rating scales, psychiatric interviews, and diagnostic assessments can be reliably conducted using VC and are generally equivalent to those performed in person. Continuing improvement in technology has mitigated many of the shortcomings found in older studies. For example, as reported in their small study, Salzman *et al*. [21] found that the only major source of disagreement on BPRS ratings between VC and in person was on patient self-neglect, which they attributed to difficulty in evaluating this construct with VC. However, Zarate *et al*. [23] found that ratings of negative symptoms were significantly improved in a high bandwidth condition as compared to a low bandwidth condition. These findings suggest that higher bandwidth and better quality equipment is associated with increased ability to observe negative symptoms and improved inter-rater reliability. Additionally, higher bandwidth leads to higher rates of acceptance and satisfaction. As both of these studies were reported over a decade ago, the vastly improved picture quality of newer VC equipment, greater accessibility of broadband connectivity, and ability to zoom and scan has made this finding significantly less of an issue. Concluding their review and meta-analysis of the literature comparing psychiatric assessments via VC to in person, Hyler *et al*. [16] opined, 'over the next few years, we expect telepsychiatry to replace [in person] in certain research and clinical situations in which the advantages outweigh the disadvantages'.

Using VC with psychotic patients has historically been met with skepticism, and rightfully so. Concerns that hallmark symptoms of the disorder including hallucinations, suspiciousness, and delusions of reference would lead patients to reject speaking with someone on a television screen are understandable, but have simply not been borne out. The primary concerns identified by patients were generally related to poor picture or audio quality. Based on a comprehensive review of the literature, there is little evidence that persons with psychosis react negatively to VC or experience exacerbations of symptoms, including patients with specific delusions involving television or being monitored. To the contrary, there is evidence that VC affords some patients a higher degree of comfort in that the perceived distance of the interaction is less anxiety provoking and reduces overstimulation found in some in person interactions [43].

The use of videoconferencing to enable remote, centralized raters in clinical trials is growing. To date, over 30,000 unique rating scale assessments have been administered to over 5,000 patients, across a range of disorders, including mood, anxiety and psychotic disorders [44]. Although there are only two published studies on the use of centralized raters in schizophrenia [41], several other trials are completed or in progress, as well as studies in other psychotic disorders. Results so far have found the methodology well accepted by patients with psychotic disorders, and that centralized ratings using VC can be conducted reliably and effectively in psychosis. Results from ongoing trials will provide additional empirical data on the use of VC in schizophrenia, as well as other disorders.

Historically, a significant concern with the feasibility of VC has been cost [45]. Although this paper was not intended to address cost, it is worth noting that, as with other areas of technology, the cost of VC equipment and connectivity, once prohibitively expensive, continues to decline. For example, Mielonen *et al*. [38] found in their analysis of cost that at a rate of 20 patients per year, the cost of VC was lower than that of the conventional alternative of traveling, and at a higher rate resulted in significant savings. They concluded that VC consultation is a cost-saving measure compared with the conventional methods requiring travel.

As noted in other literature reviews of telemedicine [46], limitations of the literature on videoconferencing and psychosis include small sample sizes, absence of control conditions, and reliance on descriptive research designs. As improvement in this technology is rapidly advancing, videoconferencing is becoming increasingly affordable, more feasible, and more widely accessible. These advances will facilitate more empirical research in this area and help guide the progress in this promising methodology.

## Competing interests

IRS, KAK and DAO are employees of MedAvante, Inc, which provides centralized ratings services via videoconferencing and rater training.

## Authors' contributions

IS conducted the literature review and drafted the manuscript. KK drafted sections of the manuscript. DO drafted sections of the manuscript. All authors read and approved the final manuscript.

## Supplementary Material

Additional file 1 **Study characteristics**. Study characteristics of articles included in the review. Click here for file
