# Supplementary material for: The use of videoconferencing with patients with psychosis: a review of the literature
Source: Ann Gen Psychiatry. 2011 Apr 18;10:14. doi: 10.1186/1744-859X-10-14 (PMC3101132; doi:10.1186/1744-859X-10-14)
Supplement: Additional file 1 — Study characteristics. Study characteristics of articles included in the review. [file 1744-859X-10-14-S1.DOC]

| Additional file 1: Study Characteristics | | | | |
| --- | --- | --- | --- | --- |
| Report Type and Author | Sample and Location | Description | Technology | Results and Comments |
| Clinical Interventions Dwyer (1973) | Mixed sample in hospital | Description of program and clinical uses | Closed Circuit Interactive (IATV) | High psychiatrist satisfaction, patients with schizophrenia had “easier” time with IATV vs. in person |
| Clinical Interventions and Satisfaction Graham (1996) | 39 patients in a community mental health setting (majority diagnosed with a major psychotic illness) | Reports on patients treated remotely using VC. | VC at 768 kbps | Decrease in hospitalizations and no sig. adverse effects reported. >90% of patients gave pos. ratings on the satisfaction survey. |
| Clinical Interventions Zaylor (1999a) | 49 patients diagnosed with either MDD or schizoaffective d/o | Retrospective review of patient records comparing clinical outcomes of patients seen by IATV | PC-based VC at 128 kbps | No sig. difference between groups. |
| Clinical Interventions Zaylor (1999b) | 6 patients with schizophrenia or schizoaffective disorder; inmates in country jail; rural group home for chronically mentally ill | Program descriptions | VC | Anecdotal report of patient improvement from VC treatment; good patient acceptance of technology; no diminution of quality of care |
| Clinical Interventions and Satisfaction Doze et al. (1999) | 109 telepsychiatry consultations were completed with 90 patients, 8 of whom were diagnosed with schizophrenia. | Results of use of VC to connect a psychiatric hospital to mental health clinics in 5 rural hospitals. | VC at 336 kbps &  384 kbps | Avoidance of hospitalization and reduced stigma for patients. Patients satisfied with and accepted experience of using VC. |
| Clinical interventions and Satisfaction  D’Souza (2000) | 28 patients: 31% diagnosed with schizoaffective disorder, 11% diagnosed with schizophreniform psychosis, and 4% diagnosed with delusional disorder. | BPRS-24 administered by both a rater familiar with the patient and a naïve rater at intake and four weeks after discharge. | Not specified | Sig. improvement in mean total BPRS scores from intake to follow-up for both raters and inter-rater reliability for the BPRS was good. Overall good satisfaction with services. |
| Clinical Interventions Kennedy and Yellowlees (2003) | 32 patients (3 diagnosed with psychotic disorders) treated by a psychiatrist via VC | Patients assessed when entering treatment and then 12 months after their initial visit. | VC at 128 kbps | Sig. improvement from pre- to post- but no significant difference between the VC and in-person condition. |
| Assessment  Dongier et al. (1986) | inpatients/outpatients; (27% schizophrenic psychoses, 6% schizophreniform psychoses, 2% paranoid states. | Compared psychiatric interviews conducted face to face and over closed circuit | Closed Circuit Television  (CCTV) | No sig differences between groups on diagnosis. Participants rated CCTV inferior to in person |

| Report Type and Author | Sample and Location | Description | Technology | Results and Comments |
| --- | --- | --- | --- | --- |
| Assessment  Yellowlees (1997) | 2 female outpatients  Diagnoses: schizoaffective psychosis and paranoid schizophrenia | Assessed patients to provide reports for a magistrate’s hearing | VC via ISDN lines at 128 kbps | Quality adequate. 1 patient w/ideas of reference from the television accepted interview as real. |
| Assessment  Ball et al. (1993) | 11 patients from an acute psychiatric ward (6 diagnosed with schizophrenia) | Compared MMSE scores from in person and over Low-Cost VideoConferencing System (LCVC) | LCVC | Scores from two conditions highly correlated (r = .89) |
| Assessment  Salzman (1996) | 6 psychotic inpatients | Compared interrater reliability on the BPRS using VC and in person | Not specified | Interrater reliability = .92. |
| Assessment and Satisfaction  Baigent et al (1997) | 63 inpatients (51% diagnosed with schizophrenia) | Compared interrater reliability on the BPRS and semi structured interview. Patients rated satisfaction with interview. | ISDN at 128 kbps | Reliability of diagnoses equivalent in 2 conditions. High acceptance for VC. |
| Assessment and Satisfaction Zarate et al. (1997) | 45 patients diagnosed with schizophrenia | Reliability of the BPRS, SAPS, and SANS in person, VC at low bandwidth (128 kbps), VC at high bandwidth (384 kbps). | VC at 128 kbps and 384 kbps | Global severity of schizophrenia and overall severity of pos. symptoms reliably assessed using VC. 384kbps more reliable than 128kbps for neg. symptoms. Good acceptance, higher bandwidth preferred by patients. |
| Assessment and satisfaction Matsuura et al. (2000) | 17 total subjects; 9 “healthy” nursing students; 8 outpatients (2 diagnosed with schizophrenia) | Examined interrater reliability on the BPRS for in person and interviews conducted by video at 2 different rates. | VC at 128 kbps and 384 kbps | ICCs were very high for VC conditions (0.965, 0.987, 0.996, respectively |
| Assessment and Satisfaction Chae et al. (2000) | 30 patients diagnosed with schizophrenia | Compared reliability on the BPRS in two conditions (15 patients in person; 15 over VC). | 33 kbps | Agreement on total BPRS for VC group significantly higher than in-person. Reliability on anxiety scale was very low for the VC group. Total acceptance scores higher for VC. |
| Assessment  Yoshino et al. (2001) | 42 patients diagnosed with chronic schizophrenia | Test-retest reliability of the BPRS in person and using two different bandwidths. | VC at 128 kbps and 2 Mbps | No sig. difference in ICC for BPRS between high bandwidth condition (0.88) and the in-person condition (0.87). ICC sig. lower in the low bandwidth condition (0.44). |

| Report Type and Author | Sample and Location | Description | Technology | Results and Comments |
| --- | --- | --- | --- | --- |
| Assessment  Lexcen et al. (2006) | 72 inpatients from a maximum security forensic unit diagnosed with schizophrenia or psychotic disorder NOS | 3 BPRS-A conditions: in-person with observation via VC, administration by VC and observation by an in-person rater, and both administration and observation in-person. | VC at 128 kbps | Correlations for total scale scores for the BPRS-A were in the good to excellent range. |
| Assessment Kobak et al. (2007) | Rater training with PANSS | Didactic and applied rater training via VC | High speed VC | VC promising for training raters to assess schizophrenia |
| Satisfaction and Acceptance Ball et al (1995) | 6 patients (three with schizophrenia and one with paranoid disorder) were included in the study. | Compared 4 different modes: in-person, telephone, hands-free telephone, and (LCVC). | LCVC | The VC condition was positively received by both patients and doctors. Some anecdotal problems were reported. |
| Satisfaction and acceptance  Mannion et al (1998) | 9 patients (two diagnosed with schizophrenia) | 8 month pilot using a PC-based VC system to facilitate emergency consultations | 384 at kbps | VC was acceptable and satisfactory for both patients and staff. |
| Satisfaction and acceptance  Stevens et al (1999) | 40 patients (19 diagnosed with psychosis) were included in the study. | 8 month pilot project of patient and clinician satisfaction with 90-minute, unstructured interviews to generate diagnoses and treatment recommendations conducted over VC or in person. | 384 at kbps | There were no differences on the patient- and clinician-rated alliance scale or the patient-rated satisfaction scale between modalities. Significant difference on the therapist version of satisfaction scale - psychiatrists tended to rate VC less favorable than in-person. Satisfaction with VC still positive. |
| Satisfaction and Acceptance  Magaletta et al. (2000) | 75 prison inmates (17 diagnosed with schizophrenia and other psychotic disorders) | Patients completed questionnaire assessing their satisfaction with receiving psychiatric consultation via VC. | 336 at kbps | Patients reported satisfaction with the consultation process, more comfort with the process over time, and a willingness to return for follow-up. |
| Satisfaction  Mielonen et al (2000) | 14 patients diagnosed with psychosis in Finland | Inpatient care-planning consultations using VC with patients and family members. Health care providers and patients and their relatives completed questionnaires of satisfaction and acceptance after each session. | unspecified | 96% of health care providers rated VC to be “as good” or “almost as good” a form of consultation as a conventional meeting. |
| Report Type and Author | Sample and Location | Description | Technology | Results and Comments |
| Clinical Trials Chen et al. (2008) | 289 subjects with schizophrenia at 35 sites | Subjects assessed weekly by one of 18 blinded, centralized raters with PANSS (1,993 assessments completed over 13 months) | VC at 384kbps | Active drug separated from placebo; no patients refused VC, high internal consistency reliability |
| Clinical Trials  Pandina et al.  (2010) | Subjects with schizophrenia | US-based subjects assessed by centralized raters in a randomized, double-blind, placebo-controlled, multicenter phase III trial | VC at 384kbps | Study had positive findings with each of the three doses of the drug demonstrating statistically significant improvement on the primary efficacy measure (PANSS total scores). Pooled data (centralized raters in U.S. and site-based raters outside of the U.S.) |
